# A Phase Field Technique for Modeling and Predicting Flow Induced Crystallization Morphology of Semi-Crystalline Polymers

**DOI:** 10.3390/polym8060230

**Published:** 2016-06-08

**Authors:** Xiaodong Wang, Jie Ouyang, Wen Zhou, Zhijun Liu

**Affiliations:** Department of Applied Mathematics, Northwestern Polytechnical University, Xi’an 710129, China; zwkakaxi@gmail.com (W.Z.); mpingke@mail.nwpu.edu.cn (Z.L.)

**Keywords:** crystallization, morphology, semi-crystalline polymer, flow induced, phase field

## Abstract

Flow induced crystallization of semi-crystalline polymers is an important issue in polymer science and engineering because the changes in morphology strongly affect the properties of polymer materials. In this study, a phase field technique considering polymer characteristics was established for modeling and predicting the resulting morphologies. The considered crystallization process can be divided into two stages, which are nucleation upon the flow induced structures and subsequent crystal growth after the cessation of flow. Accordingly, the proposed technique consists of two parts which are a flow induced nucleation model based on the calculated information of molecular orientation and stretch, and a phase field crystal growth model upon the oriented nuclei. Two-dimensional simulations are carried out to predict the crystallization morphology of isotactic polystyrene under an injection molding process. The results of these simulations demonstrate that flow affects crystallization morphology mainly by producing oriented nuclei. Specifically, the typical skin-core structures along the thickness direction can be successfully predicted. More importantly, the results reveal that flow plays a dominant part in generating oriented crystal morphologies compared to other parameters, such as anisotropy strength, crystallization temperature, and physical noise.

## 1. Introduction

For semi-crystalline polymers, the ultimate properties of the products fabricated by freezing liquids into solids strongly depend on the features of crystalline structures formed during solidification [1]. Therefore, it is crucial to understand the formations of various crystallization morphologies during processing by experimental methods or/and numerical simulations. The ultimate aim is to be able to predict the final crystalline structures and, from that, to predict the final properties of the products. Then these predictions are expected to supply the theoretical basis for controlling or optimizing the processing conditions accordingly.

During polymer processing, such as injection molding and fiber spinning, polymer liquids often undergo shearing or stretching of the forced flow field. In this situation, the crystallization behaviors of semi-crystalline polymers will change greatly compared to the crystallizations in quiescent conditions. It is often believed that flow can shorten the nucleation time, enhance the nucleation density, and accelerate the crystal growth of polymers. Besides, flow can also change the crystallization morphologies of polymers, and produce more plentiful crystal patterns (such as columnar crystals, shish-kebab crystals, and fibrillar crystals) than quiescent conditions [2].

Since the pioneering work of Mitsuhashi on flow induced crystallization in 1963 [3], a substantial research effort has been conducted. So far, researchers have obtained quite a number of important achievements on this topic [4,5,6,7]. However, due to the limitations of experimental conditions and lack of appropriate experimental methods, there are still many issues to be addressed. Over these years, with the development of the computer and a wide range of application of simulation technologies, simulation has played an important role in understanding flow induced crystallization. Generally, the study of polymer crystallization under flow conditions consists of three aspects, which are, kinetics, rheology, and morphology. Simulation studies on kinetics and rheology have been carried out for a long time, and a number of valuable theories and models have been built [8,9,10]. However, researching the flow induced crystallization morphology by means of numerical simulation is still a tough road to walk. The major challenges are how to establish reasonable and computable models, and propose effective solving algorithms accordingly. For this reason, this paper is devoted to the modeling and predication of flow induced crystallization morphology of semi-crystalline polymers.

At the moment, there are several kinds of methods may be used to simulate the crystallization morphology of semi-crystalline polymers. These include cellular automaton methods [11,12], geometric methods based on kinetics [13,14], phenomenological methods based on kinetics [15,16], molecular dynamics or Monte-Carlo methods [17,18,19,20,21], interface capturing methods [22,23,24], phase field methods [25,26,27,28], *etc.* The cellular automaton methods need to set the rules of crystal growth in advance. Because the isotropic growth rules of spherulite are very simple, this kind of method is mainly used for simulating crystallization morphology in quiescent conditions. However, for flow induced crystallization morphology, because of its complex growth rules, cellular automaton methods are difficult to generalize for that case. Geometric methods are simple to use. Whereas, due to the spherical crystal structure assumption, they are not suitable for the flow induced case. Phenomenological methods can provide some statistical information of flow induced crystallization morphology, but cannot visualize the results. Although molecular dynamics or Monte-Carlo methods can obtain some information of nucleation and crystal growth, the simulated spatial and temporal scale, which is subject to the computing capacity of the modern computer, is very limited. Interface capturing methods are still in early stages of development and simulated crystallization morphologies are rather few. By contrast, phase field methods can simulate many crystallization morphologies, which are consistent with experimental results. Among these methods, we think that phase field methods have more potential to be generalized to simulate flow induced crystallization morphology.

Early on, phase field methods were mainly used to simulate the crystal growth of metals and small molecular compounds. Nevertheless, in recent years, researchers have generalized this kind of method to simulation of polymer crystallization [25]. These methods directly use a group of nonlinear partial differential equations to describe the complex crystal growth of a polymer. This makes the phase field methods able to simulate very rich crystallization morphologies and microstructures with moderate computing cost [28]. Therefore they become promising methods to simulate the crystallization morphology of a polymer. However, since flow effects have not been considered for the models in published literature, at present phase field methods for polymer crystallization can only be used for quiescent conditions. Therefore, the primary work of this paper is to develop a phase field technique for polymer crystallization under flow induced conditions. 

In fact, there have been many studies coupling phase field with flow field in metal and small molecular compound crystallization [29,30]. However, because the crystallization of polymers under flow conditions is more complex, these studies cannot be simply generalized to the case of polymer crystallization. In the presence of flow, it is well believed that the polymer chain segments in the melt will be extended from random coil conformation into microfibrillar structure along the flow direction [31]. These ordered structures are usually known as precursors. In this work, such precursors are produced by flow treatment prior to the start of crystallization. Moreover, it has been demonstrated that flow induced precursors could survive a long time at high temperature [32,33]. After the cessation of flow, oriented nuclei are easily formed depending on the precursors. Furthermore, these nuclei can remarkably change the crystal growth of semi-crystalline polymers during the subsequent crystallization, which is referred to as “memory effect” [33]. Therefore, in modeling crystalline morphologies produced by flow, this paper will first establish a flow induced nucleation model with the aid of calculable flow induced molecular orientation and stretch, then predict the subsequent crystal growth via a modified phase field method.

The rest of this paper is organized as follows. In Section 2, we attempt to present a quantitative description of flow induced nucleation by using calculated information of molecular orientation and stretch based on the FENE-P (Finitely Extensible Nonlinear Elastic dumbbell model with Peterlin closure) model. Section 3 gives the theoretical description of our phase field technique for modeling the crystal growth and then, in Section 4, the proposed model is used to simulate the crystallization morphology of isotactic polystyrene with the injection molding process which can be simplified as a plane Poiseuille flow in 2D. Finally, some brief conclusions are drawn in Section 5.

## 2. Modeling Nucleation

In this section, a nucleation model based on flow induced structures is established. In this model, the flow induced structures including molecular orientation and stretch are described by the FENE-P model, where the variable of orientational tensor contains information on molecular orientation and stretch. Hence, the orientation and aspect ratio of a flow induced nucleus, which is assumed to possess a cylindrical appearance for the sake of simplicity, may be determined by the orientational tensor. Coincidentally, the above idea is very similar to the recently proposed approach by Pantani *et al.* [34]. Although the two methods are different, their feasibility may be confirmed by comparing with each other. Details of this model are shown below.

### 2.1. FENE-P Model for Describing Molecular Orientation and Stretch

Today, several computational models are available for predicting molecular orientation and stretch of a polymer undergoing flow, such as the Leonov model [35], the FENE-P model [36], *etc.* In view of the wide and mature application of the FENE-P model, this is adopted in this work.

**Figure 1:**
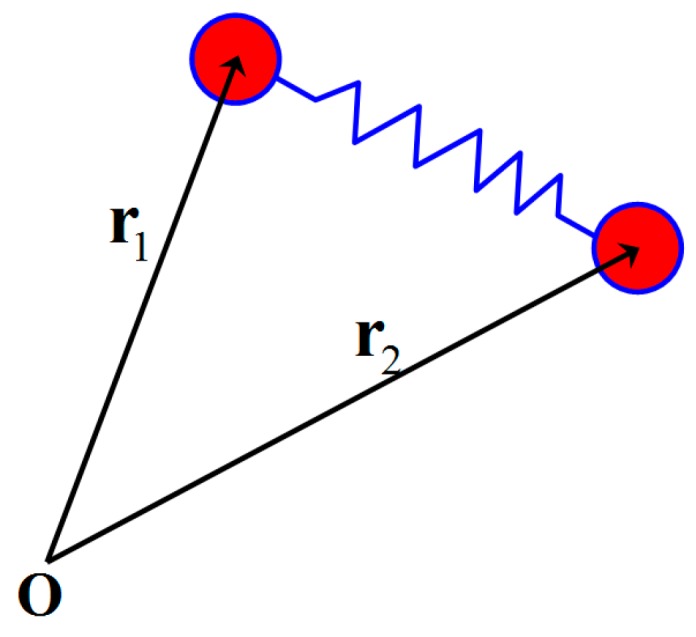
Schematic diagram of the elastic dumbbell. The dumbbell consists of two rigid balls with mass *m* and position vectors **r**_1_ and **r**_2_, where the balls are connected by a massless spring and **O** is the coordinate origin.

The FENE-P model was developed from the dumbbell model of polymer molecules. It was first used to describe the rheological behavior of a dilute polymer solution, and then expanded to concentrated polymer solutions and even polymer melts. Figure 1 shows a schematic diagram of an elastic dumbbell, which is an abstract representation of a polymer molecule. At any given time, the balls of a dumbbell are subjected to friction force, Brownian force, and spring force. According to Newton’s second law, kinematic equations of the balls can be obtained

(1) $$m\frac{d^{2}r_{i}}{dt^{2}}=-\varsigma (\frac{dr_{i}}{dt}-u(r_{i}))-k_{B}T\frac{\partial }{\partial r_{i}}\ln\varphi +F_{i},\;(i=1,\;2)$$

where ς is the friction coefficient and **u** (**r***_i_*) = **u**_0_ + (∇**u**)*^T^***r***_i_* is the velocity of solution at position **r***_i_*. The terms *k*_B_, *T*, and ϕ are the Boltzman constant, thermodynamic temperature, and probability density function of the dumbbell distribution, respectively. The spring force **F** = **F**_1_ – **F**_2_ as they are a pair of action and reaction. Because the acceleration on the left hand side usually can be neglected, by subtracting the two equations in Equation (1) we get

(2) $$\frac{dQ}{dt}={\left(\nabla u\right)}^{T}\cdot Q-\frac{2k_{B}T}{\varsigma }\frac{\partial }{\partial Q}\ln\varphi -\frac{2}{\varsigma }F$$

where **Q** = **r**_2_ − **r**_1_ is the end-to-end vector of the dumbbell. 

On the other hand, ϕ should satisfy the following continuity equation based on Fick’s law,

(3) $$\frac{\partial \varphi }{\partial t}+\frac{\partial }{\partial Q}\cdot \left(\frac{dQ}{dt}\varphi \right)=0$$

Inserting Equations (2) to (3), a Fokker-Planck equation for ϕ is obtained as

(4) $$\frac{\partial \varphi }{\partial t}+\frac{\partial }{\partial Q}\cdot \left({\left(\nabla u\right)}^{T}\cdot Q\varphi \right)-\frac{2k_{B}T}{\varsigma }\frac{\partial^{2}\varphi }{\partial Q^{2}}-\frac{2}{\varsigma }\frac{\partial }{\partial Q}\cdot \left(\varphi F\right)=0$$

Generally, directly solving Equation (4) is rather difficult, so the moment-closure approach is used in this paper. In a typical derivation of the moment-closure equations, one multiplies the Fokker-Planck equation by certain powers of **Q**, and integrates over the conformation space. Therefore, for the Fokker-Planck Equation (4) we obtain the following equation of motion for the second moments:

(5) $$\frac{D\langle QQ\rangle }{Dt}-{\left(\nabla u\right)}^{T}\cdot \langle QQ\rangle -\langle QQ\rangle \cdot (\nabla u)=\frac{4k_{B}T}{\varsigma }I-\frac{4}{\varsigma }\langle QF\rangle$$

where *D/Dt*=∂/∂*t*+**u∙**∇ is the material derivative, **I** is the unit tensor, and the second moments <**QQ**> represents the conformation tensor. For the FENE-P model, the spring force in Equation (5) is approximated as

(6) $$F=\frac{HQ}{1-\langle Q^{2}/Q_{0}^{2}\rangle }$$

where H is the elastic coefficient, *Q* = |**Q**| is the length of spring, *Q*_0_ is the maximum extensibility. By inserting Equations (6) into (5) and defining the dimensionless conformation tensor **C** = *H*<**QQ**>/*k*_B_*T*, we get the following evolution equation:

(7) $$\lambda \left(\frac{DC}{Dt}-{\left(\nabla u\right)}^{T}\cdot C-C\cdot (\nabla u)\right)=-\frac{C}{1-tr(C)/b}+I$$

where *b* = $HQ_{0}^{2}$/*k*_B_*T* is the dimensionless maximum extensibility and λ = ζ/4*H* is the molecular relaxation time. According to the dimensionless conformation tensor **C**, information of the molecular orientation and stretch can be obtained. Details of this are discussed in Section 2.3. Besides, the extra stress of polymer can be calculated by the Giesekus expression

(8) $$\tau_{p}=\frac{\eta_{p}}{\lambda }\left(\frac{C}{1-tr(C)/b}-I\right)$$

where η_p_ is the viscosity of polymer.

### 2.2. FENE-P Flow Model for Describing the Macroscopic Flow Field

A general FENE-P model in the background of macroscopic flow field can be described by coupling Equations (7) and (8) with the incompressible Navier-Stokes equations

(9) ∇⋅u=0

(10) $$\rho (\frac{\partial u}{\partial t}+u\cdot \nabla u)=-\nabla p+\eta_{s}\Delta u+\nabla \cdot \tau_{p}+b$$

where ρ, *p*, η*_s_*, and **b** are the density, pressure, viscosity of solvent, and body force per unit volume, respectively. By introducing the characteristic length for single crystals *d*, the mass diffusivity *D* and total viscosity of polymer η = η_s_ + η_p_, and defining dimensionless variables

$$t^{*}=\frac{D}{d^{2}}t,\;x^{*}=\frac{x}{d},\;u^{*}=\frac{d}{D}u,\;p^{*}=\frac{d^{2}}{\eta D}p,\;\tau_{p}^{*}=\frac{d^{2}}{\eta D}\tau_{p},\;b^{*}=\frac{d^{3}}{\eta D}b$$

and dimensionless parameters

$$\beta =\frac{\eta_{s}}{\eta_{s}+\eta_{p}},\;Re=\frac{\rho D}{\eta },\;We=\lambda D/d^{2}$$

the following dimensionless coupled system (the superscript is omitted) is obtained:

(11) ∇⋅u=0

(12) $$Re(\frac{\partial u}{\partial t}+u\cdot \nabla u)=-\nabla p+\beta \Delta u+\nabla \cdot \tau_{p}+b$$

(13) $$We\left(\frac{DC}{Dt}-{\left(\nabla u\right)}^{T}\cdot C-C\cdot (\nabla u)\right)=-\frac{C}{1-tr(C)/b}+I$$

(14) $$\tau_{p}=\frac{1-\beta }{We}\left(\frac{C}{1-tr(C)/b}-I\right)$$

Here, the dimensionless parameter β represents the contribution rate of solvent viscosity to the total viscosity, the Reynolds number *Re* is the ratio of momentum forces to viscous forces quantifying the relative importance of these two types of forces for given flow conditions, and the Weissenberg number *We* compares the viscous forces to the elastic forces.

As is well known, numerical difficult usually arises when solving the coupled system (11)–(14). That is, the so called “high Weissenberg number problem” [37]. In order to avoid this kind of difficulty, only the plane Poiseuille flow with steady-state solution is considered. If we assume that the flow always reaches its steady-state during the induced period, then the final molecular conformation caused by such flow will be solved exactly.

**Figure 2:**
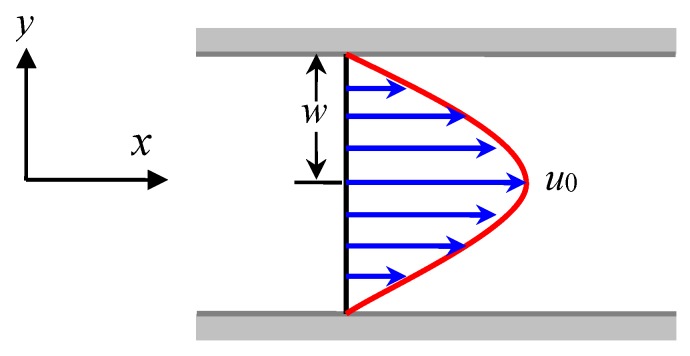
Schematic diagram of the steady-state plane Poiseuille flow. The blue arrows show the flow direction as well as the corresponding strength, the red curve represents the velocity profile, *w* and *u*_0_ are respectively the half width of the tube and middle axis velocity.

For the steady-state plane Poiseuille flow illustrated in Figure 2, its velocity can be expressed as **u** = (*u*, *v*)*^T^* = (*u*_0_(1 − *y*^2^/*w*^2^), 0)*^T^*, where *u*_0_ is the velocity at the middle axis and *w* is the half width of the tube. Because the flow is in its steady-state and the flow variables are invariant along the *x*-axis, the conformation tensor **C** is irrelevant to *t* and *x*. Therefore, we have

(15) $$\frac{DC}{Dt}-{\left(\nabla u\right)}^{T}\cdot C-C\cdot (\nabla u)=\frac{\partial C}{\partial t}+(u\cdot \nabla )C-{\left(\nabla u\right)}^{T}\cdot C-C\cdot (\nabla u)=-\gamma \left(\begin{array}{cc} 2C_{12} & C_{22}\\ C_{22} & 0 \end{array}\right)$$

where γ = ∂*u*/∂*y* = −2*u*_0_*y*/*w*^2^. By inserting Equations (15) into (13), we get

(16) $$\gamma We\left(\begin{array}{cc} 2C_{12} & C_{22}\\ C_{22} & 0 \end{array}\right)=\frac{C}{1-tr(C)/b}-I$$

that is

(17) $$2\gamma WeC_{12}=C_{11}/(1-tr(C)/b)-1$$

(18) $$\gamma WeC_{22}=C_{12}/(1-tr(C)/b)$$

(19) $$C_{22}/(1-tr(C)/b)-1=0$$

From Equations (17)–(19), it is easy to get

(20) $$C=\frac{b}{b+2}I$$

for *y* = 0 and

(21) $$C_{22}=\frac{|\gamma |We\sqrt{6(b+2)}\left(K-\frac{1}{K}\right)}{6{\left(\gamma We\right)}^{2}}$$

(22) $$C_{11}=b-(b+1)C_{22}$$

(23) $$C_{12}=\gamma WeC_{22}^{2}$$

for *y* ≠ 0, where

(24) $$K=\sqrt[{3}]{\delta +\sqrt{\delta^{2}+1}}$$

(25) $$\delta =\frac{54b|\gamma |We}{{\left(\sqrt{6(b+2)}\right)}^{3}}$$

If we insert γ = −2*u*_0_*y*/*w*^2^ into Equations (21)–(23), then the conformation tensor under the plane Poiseuille flow will be exactly solved.

### 2.3. Flow Induced Structures

**Figure 3:**
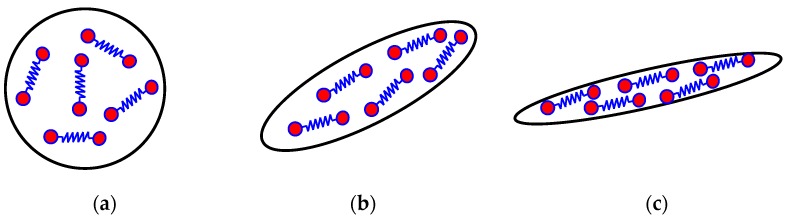
Schematic diagrams for conformation transformation of the macromolecules by flow: (**a**) conformation sphere without flow; (**b**) conformation ellipsoid at weak flow; (**c**) conformation pipe at strong flow.

Under the shear and elongation of an applied flow field, ordered structures can be generated from the originally disordered polymer melts or solutions. From the perspective of polymer rheology, ordered structures are generally characterized by molecular orientation and stretch, which can be described by the conformation tensor in the framework of dumbbell models. Figure 3 illustrates the conformation transformation under flow. As can be seen, the conformation transformation is very similar to the molecular deformation under flow.

From the definition **C** = *H*<**QQ**>/*k_B_T*, it is obvious that **C** is a symmetric positive definite second-rank tensor. This ensures that we can always get the eigenvalues and eigenvectors of the conformation tensor. If we regard the directions of the eigenvectors as the major and minor axes and the eigenvalues as the corresponding lengths, then an orientation ellipse for 2D or an orientation ellipsoid for 3D will be obtained. Specifically, when the polymer is in its equilibrium state (without flow), we have **C** = *b*/(*b* + *d*_s_)**I**, where *d*_s_ = 2, 3 is the dimension of space. In this situation, the orientation ellipse or ellipsoid degenerates to orientation circle or sphere, which means the orientation probability along any direction is the same. Otherwise, the orientation along the major axis will be dominant. In this work, we simply deem that the molecule is always oriented along the major axis of the orientation ellipse or ellipsoid when it is undergoing flow. In simulations, the eigenvalues and eigenvectors of **C** in 2D can be calculated by

(26) $$\lambda_{1,\;2}=\frac{tr(C)\pm \sqrt{{\left(tr(C)\right)}^{2}-4det(C)}}{2}$$

(27) $$\xi_{1,\;2}={\left(1,\;\frac{\lambda_{1,2}-c_{11}}{c_{12}}\right)}^{T}$$

for *c*_12_ ≠ 0 and

(28) $$\lambda_{1}=c_{11},\;\lambda_{2}=c_{22}$$

(29) $$\xi_{1}={\left(1,\;0\right)}^{T},\;\xi_{2}={\left(0,\;1\right)}^{T}$$

for *c*_12_ = 0.

Besides the molecular orientation, the conformation tensor may also be used to quantify the molecular stretch. Generally, the first invariant *tr*(**C**) is treated as a measure of molecular stretch. In equilibrium state, we have *tr*(**C**) = 2*b*/(*b* + 2) for 2D. In non-equilibrium state, the deformation of the macromolecular chain will increase as the shear or elongation is intensified, and accordingly *tr*(**C**) will trend to the maximum extensibility *b*. In practice, the amount of molecular stretch is an intermediate cause of anisotropic nucleation, and hence the cause of the anisotropic crystallization morphology. So, *tr*(**C**) should be considered in modeling the nucleation and crystal growth of flow induced polymer crystallization.

### 2.4. Oriented Nucleus

Flow affects the crystallization of the polymer by providing both extra nucleation sites and oriented templates. The former accelerates the kinetics of the crystallization and the latter induces anisotropy to the crystalline morphology. Because of experimental observations, the number of activated nuclei may be written as the sum of the general nuclei observed in quiescent condition *N*_q_, and the additional nuclei appearing after flow *N*_f_, *viz*,

(30) $$N=N_{q}+N_{f}$$

Based on the work of Koscher and Fulchiron [38], the number of additional nuclei appearing after flow treatment is linked to the first normal stress difference *N*_1_. The simplest mathematical relationship between *N*_f_ and *N*_1_ is

(31) $${\dot{N}}_{f}=CN_{1}$$

where *C* is a scale factor determined from experiments. In quiescent conditions, the following equation is reported in the literature describing the nucleation density as a function of temperature

(32) $$N_{q}(T)=N_{0}\exp\left(\vartheta \cdot (T_{m}-T)\right)$$

where *T*_m_ is the melting temperature, *N*_0_ and ϑ are empirical parameters. For 2D problems, the 3D nucleation density giving in Equation (32) should be converted to a 2D nucleation density. Referring to the work done by Charbon and Swaminarayan [39], the stereological relationship

(33) $$N_{2D}=1.458{\left(N_{3D}\right)}^{2/3}$$

can be adopted. Since the influence of flow on the growth rate is less relevant than on the nucleation process [40], the considered effect of flow on crystallization kinetics is limited to the above.

Besides, another function of flow is that it helps to generate the oriented nucleus. In theoretical modeling, the oriented nucleus is usually considered to possess a cylindrical appearance [41]. Thus, it can be described by two characters, which are orientation and aspect ratio. To keep it simple, we assume that the ordered structures induced by flow would be completely retained after the cessation of flow until the nucleation takes place. Based on this assumption, the orientation of one nucleus is directly set as the molecular orientation where it is located. And accordingly, the aspect ratio of the nucleus, which is associated with the amount of the molecular stretch *tr*(**C**), is determined by the following equation:

(34) $$l_{w}=1+M\sqrt{1-{d_{s}}^{d_{s}}det\left(\frac{C}{tr(C)}\right)}\;tr(C)$$

where the coefficient *M* depends on the crystallization property of the polymer. Specially, the above equation in 2D can be written as

(35) $$l_{w}=1+M\sqrt{1-\frac{4det\left(C\right)}{{\left(tr(C)\right)}^{2}}}\;tr(C)=1+M\left|\lambda_{1}-\lambda_{2}\right|$$

which means that ∆ = λ_max_ − λ_min_ can be regarded as another measure of molecular stretch. Recently, this measure was adopted by Pantani *et al.* in modeling morphology evolution during polymer crystallization under processing conditions [34].

In the studies based on phase field methods, researches mainly considered the point-like nuclei. However, in our previous work, we developed the oriented nuclei into phase field modeling [42]. In this study, the nuclei used in simulation are more complicated. Their shapes are dependent on the flow history prior to nucleation events. Point-like and oriented nuclei may appear together in one simulation. To generate a nucleus, we should first figure out its orientation and aspect ratio according to the flow history at the position where the nucleus is located, and then “create” it on the lattice by using the method given in our early work [42].

## 3. Modeling Crystal Growth

In this section, a phase field method for modeling the crystal growth of a polymer will be briefly introduced. Because the effect of flow prior to crystallization on crystal growth is less relevant than on nucleation [40], the crystal growth model does not involve flow effect.

### 3.1. Free Energy of the System

In phase field modeling, the total free energy of the system *F*(ψ, *T*) consists of two parts, which are a local free energy density *f_local_*(ψ, *T*) and a non-local free energy density *f_grad_*(ψ), *viz*,

(36) $$F(\psi ,T)=\int \left[f_{local}(\psi ,T)+f_{grad}(\psi )\right]d\Omega$$

where *T* is the temperature, ψ is a non-conserved crystal order parameter, and Ω is the region occupied by the system. Following the work by Kyu and coworkers [25], ψ is defined as the ratio of lamellar thickness over optimum lamellar thickness and its value indicates whether the substance is liquid or solid. Specifically, 0 represents the melt and 1 the crystal at equilibrium.

For polymer crystallization, the local free energy density is given in the form of an asymmetric double well as follows [28]:

(37) $$f_{local}(\psi ,T)=W\int_{0}^{\psi }\phi (\phi -\zeta_{0})[\phi -\zeta (T)]d\phi$$

where *W* describes the height of the energy barrier for surface nucleation, ζ(*T*) is the unstable energy barrier, and ζ_0_ is the value of ψ relevant to the stable solidification potential. In simulations, ζ_0_ may be simply estimated as ζ_0_ = *T*_m_/$T_{\text{m}}^{0}$, where *T*_m_ and $T_{\text{m}}^{0}$ are respectively the experimental melting temperature and equilibrium melting temperature. The nonlocal free energy density describing the symmetric or asymmetric growth process can be written as

(38) $$f_{grad}(\psi )=\frac{1}{2}\kappa_{0}^{2}\beta^{2}(\theta ){\left(\nabla \psi \right)}^{2}$$

where *κ*_0_ is the coefficient of interface gradient, θ is the orientation angle, and β(θ) describes the anisotropic growth rate of the interface. In the literature, β(θ) is often given as

(39) β(θ)=1+εcos(jθ)

with ε is the strength of anisotropy and *j* the number of mode. Here, θ is defined as the angle between the interface normal and the reference axis, *i.e.*,

(40) $$\theta =\text{arctan}(\psi_{y}/\psi_{x})$$

### 3.2. Phase Field Model

According to the Ginzburg-Landau theory, the temporal evolution of ψ can be governed by

(41) $$\frac{\partial \psi (x,t)}{\partial t}=-\Gamma \frac{\delta F(\psi ,T)}{\delta \psi (x,t)}$$

where Γ is the rotational mobility of the system. By substituting Equations (36)–(38) into (41), one obtains the following phase field equation for 2D:

(42) $$\begin{array}{l} \frac{\partial \psi (x,t)}{\partial t}=-\Gamma (W\psi (\psi -\zeta (T))(\psi -\zeta_{0})-\kappa_{0}^{2}\nabla \cdot \left(\beta^{2}(\theta )\nabla \psi \right)\\ \;+\kappa_{0}^{2}\frac{\partial }{\partial x}\left(\beta (\theta )\beta^{\prime }(\theta )\frac{\partial \psi }{\partial y}\right)-\kappa_{0}^{2}\frac{\partial }{\partial y}\left(\beta (\theta )\beta^{\prime }(\theta )\frac{\partial \psi }{\partial x}\right)) \end{array}$$

To determine the temperature at the growing crystal fronts, a heat conduction equation may be deduced from the conservation law of enthalpy taking the form of

(43) $$\frac{\partial T}{\partial t}=\alpha \nabla^{2}T+K\frac{\partial \psi }{\partial t}$$

where α = *k_T_*/(ρ*C*_p_) and *K* = Δ*H*/*C*_p_ with *C*_p_ and *k*_T_ being the specific heat capacity and thermal conductivity, respectively. The coupled system of Equations (42) and (43) is just our phase field model.

By using the dimensionless variables defined in Section 2.2 and rescaling the temperature to *U* = (*T* − *T*_c_)/(*T*_m_ − *T*_c_) with *T*_c_ being the experimental temperature of crystallization, the phase field model can be represented in the following dimensionless form:

(44) $$\begin{array}{l} \frac{\partial \psi }{\partial t}=-(W\psi (\psi -\zeta )(\psi -\zeta_{0})-{\tilde{\kappa }}_{0}^{2}\nabla \cdot \left(\beta^{2}(\theta )\nabla \psi \right)\\ \;+{\tilde{\kappa }}_{0}^{2}\frac{\partial }{\partial x}\left(\beta (\theta )\beta^{\prime }(\theta )\frac{\partial \psi }{\partial y}\right)-{\tilde{\kappa }}_{0}^{2}\frac{\partial }{\partial y}\left(\beta (\theta )\beta^{\prime }(\theta )\frac{\partial \psi }{\partial x}\right)) \end{array}$$

(45) $$\frac{\partial U}{\partial t}=\tilde{\alpha }\nabla^{2}U+\tilde{K}\frac{\partial \psi }{\partial \tau }$$

where ${\tilde{\kappa }}_{0}^{2}=\kappa_{0}^{2}/d^{2}$, $\tilde{\alpha }=\alpha /D$, $\tilde{K}=K/\left(T_{m}-T_{c}\right)$, and the mobility Γ is estimated from *d* and *D* as Γ = *D*/*d*^2^.

In practice, the model parameters *W* and *κ*_0_ can be determined through experimentally measurable quantities. They may be calculated by [25]

(46) $$W=6\frac{\Delta H}{RT\zeta_{0}^{3}}\left(\frac{T_{m}-T}{T_{m}^{0}}\right){\left(\frac{\zeta_{0}}{2}-\zeta \right)}^{-1}$$

(47) $$\kappa_{0}=6\frac{\sigma }{nRT}{\left(\frac{2}{W}\right)}^{1/2}$$

with Δ*H* being the latent heat, *R* the gas constant, σ the surface free energy per unit area, and *n* the amount of substance of polymer monomers per unit volume. Finally, the unstable energy barrier ζ, which increases with temperature, may be determined by [28]

(48) $$\zeta =[1+\frac{2}{\pi }\text{arctan}(kU)]\hat{\zeta }$$

(49) $$\hat{\zeta }=\frac{4\zeta_{0}\hat{\psi }-3{\hat{\psi }}^{2}}{6\zeta_{0}-4\hat{\psi }}$$

(50) $$\hat{\psi }=\frac{T_{m}^{0}-T_{m}}{T_{m}^{0}-T}\zeta_{0}$$

where k is a dimensionless coefficient inversely proportional to the supercooling. In this work, it is simply evaluated as $k=\tilde{K}$.

## 4. Results and Discussion

The complete research of flow induced crystallization should cover both the nucleation and crystal growth processes. Because nuclei can form quite rapidly and to achieve *in-situ* tracking of their formation puts severe demands on the experimental methods used, most of the previous studies focus on the crystallization after cessation of flow [43]. The physical problem we considered was first producing the flow of hot polymer and then cooling it quite rapidly after cessation of flow. In the process, nucleation takes place during or immediately after flow treatment, while crystal growth starts from the cessation of flow. For this reason, the simulations may be divided into three steps. The first step is to predict the molecular conformation by simulating the viscoelastic flow in the framework of the dumbbell model; the second step is to generate oriented nuclei dependent on the flow induced structures; and the last step is to simulate the crystal growth via the phase field method.

Simulations are carried out in 2D for the models presented above. The simulated case is the crystallization of isotactic polystyrene under the injection molding process which can be simplified as a plane Poiseuille flow in 2D (shown in Figure 2). For the flow field, the dimensionless parameters used in simulations are *w* = 512 and *b* = 200. In order to facilitate the following discussion, the maximum shear rate of the plane Poiseuille flow is denoted by γ_m_ = 2*u*_0_/*w*, and a dimensionless parameter $\hat{\gamma }=We\cdot \gamma_{m}$ is used to measure the relative flow strength. For the crystallization, the model parameters are calculated using a set of experimentally accessible physical parameters of isotactic polystyrene (iPS) at a crystallization temperature of *T*_c_ = 200 °C (If not otherwise specified, the simulations are done at this temperature by default). These parameters include $T_{\text{m}}^{0}$ = 242 °C, ζ_0_ = 0.953, ζ = 0.167, W = 15.43, ${\tilde{\kappa }}_{0}^{2}=0.916$, $\tilde{\alpha }=0.658$ and $\tilde{K}=1.578$. It should be noted that the above parameters are dependent on the crystallization temperature. To obtain other sets of model parameters for crystallization, the reader can refer to previously published papers [44,45] for details. Besides, the empirical parameters for nucleation are assigned values *N*_0_ = 1.74 × 10^12^/m^3^ and ϑ = 0.155 [46].

**Figure 4:**
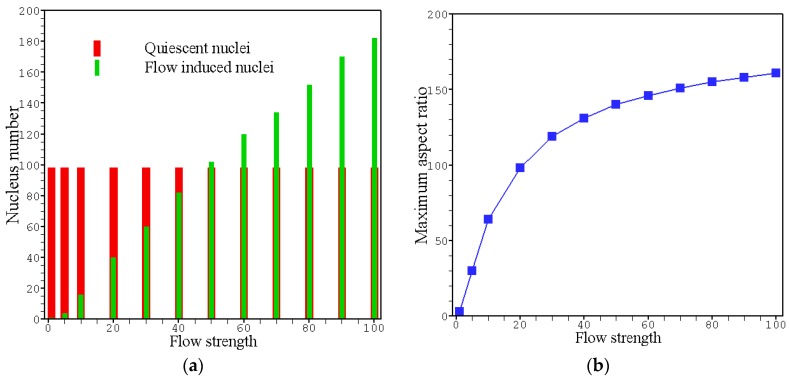
Flow effects on nucleus number and nucleus shape within the simulation region: (**a**) the variation of nucleus number (refer in particular to the flow induced part) with the dimensionless flow strength $\hat{\gamma }$; (**b**) plot of the maximum aspect ratio of the nuclei against the dimensionless flow strength $\hat{\gamma }$.

In our nucleation model, the nucleus number and nucleus shape are both influenced by flow. As can be seen from Figure 4, the nucleus number (quiescent nuclei add flow induced nuclei) increases with flow strength. This prediction agrees very well with most experimental observations [38], and has been regarded as the first noticeable effect of flow treatment. Specifically, under weak flow treatment, quiescent nucleation dominates. However, when the flow is strong enough, flow induced nucleation will surpass quiescent nucleation as a leading part. As to the nucleation shape, oriented nuclei or thread-like precursors have already been observed in experiments [47], and the aspect ratios of these nuclei are dependent on the strength of applied flow treatment. In this study, because the shear rate is not constant over the whole flow field, only the maximum aspect ratio of the oriented nuclei is shown in Figure 4. Following the increase of flow strength, the maximum aspect ratio of the nuclei increases gradually but the increase rate tends to be reduced. This phenomenon is in accordance with the fact that the degree of segmental orientation of the macromolecule increases but does not immeasurably increase with flow. This means that our nucleation model is able to predict the formation of oriented nuclei in the sample. Besides, the aspect ratios of nuclei should be also dependent on the chain structure of the polymer. For example, polymers consisting of linear and long chains are easier to be stretched and oriented under the same flow treatment. In our nucleation model, the effect from the chain structure of the polymer itself is connected with the dimensionless maximum extensibility *b*.

**Figure 5:**
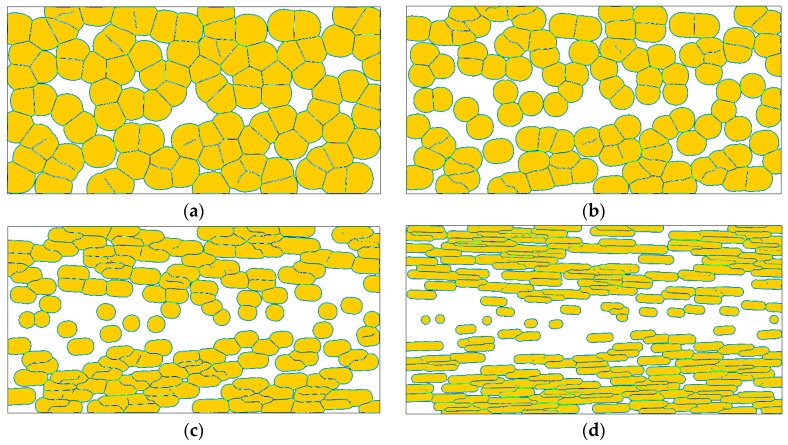
Simulated crystallization morphologies in the crystal order parameter field for different flow strength without considering the interface anisotropy (*i.e.*, ε = 0): (**a**) $\hat{\gamma }=1.0,\;t=800$; (**b**) $\hat{\gamma }=10,\;t=600$; (**c**) $\hat{\gamma }=30,\;t=400$; (**d**) $\hat{\gamma }=100,\;t=200$. Here the time instants are chosen to ensure the results reach similar sizes of crystallized regions.

It is foreseeable that the changes of nucleation caused by flow treatment will certainly affect the later crystal growth. Figure 5 shows the crystallization morphologies obtained by our model for a set of flow strengths. In carrying out these simulations, the anisotropic growth of the crystal interface was not taken into consideration, *i.e.*, ε = 0. As has been discussed in the modeling sections, influence of flow on polymer crystallization is twofold, one is on morphology and the other is on kinetics. Because our attention was mainly focused on the morphology, the results shown in each figure are adapted to ensure similar sizes of crystallized regions for comparison. From Figure 5, it is evident that flow has important influence on the crystallization morphology. Under weak flow treatment (Figure 5a), spherulites are obtained throughout the simulation region, and the results show almost no difference with those obtained under quiescent conditions [28]. As the flow strength is intensified, the spherulites near the skin layer in Figure 5b begin to present elliptical outlines, and in Figure 5c the morphology is much different to that shown in Figure 5a. Further, in Figure 5d the morphology is transformed from spherulites to oriented crystals. In the literature, similar results were obtained from experimental observations [38,48].

**Figure 6:**
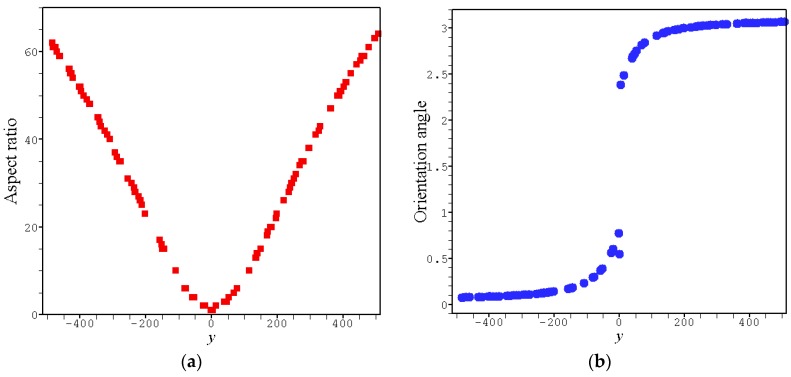
Distribution of the oriented nuclei along the *y*-axis obtained for $\hat{\gamma }=10$, where each symbol represents a nucleus: (**a**) aspect ratio; (**b**) orientation angle.

Specifically, the orientation degree near the skin layer is greater than that close to the core layer. The main reason for this is obviously that the flow induced nucleation changes along the vertical direction of the channel due to the change of shear rate. For a quantitative observation, Figure 6 gives the distribution of oriented nuclei obtained for the flow strength $\hat{\gamma }=10$. It is clear that the nuclei near the skin layer have greater aspect ratios and their orientations are closer to the flow direction. Also, the symbol distribution in the figure indicates that the nucleus density near the skin layer is greater than the core layer, which directly causes the faster crystallization near the skin layer. Therefore, in real processing, the lower temperature near the skin layer (compared to the core layer) is not the only reason for faster crystallization.

In addition, from Figure 5, the crystallization after stronger flow treatment needs less time to reach a similar size of crystallized region. This means that the crystallization after stronger flow treatment is faster than that after weaker flow treatment [49]. In this study, the flow treatment is applied for a short time and the subsequent crystallization actually occurs in quiescent conditions. That is, the flow does not show any direct increase in crystal growth rate. Therefore, the faster crystallization after stronger flow treatment is mainly caused by the fact that the flow can remarkably enhance the nucleation rate. Another phenomenon caused by flow induced nucleation is the change in size of the crystals [50]. This can be clearly seen in Figure 5a,b. It is obvious that the crystals in Figure 5b have smaller sizes, and an explanation of this is that more activated nuclei usually result in a narrower growing space.

**Figure 7:**
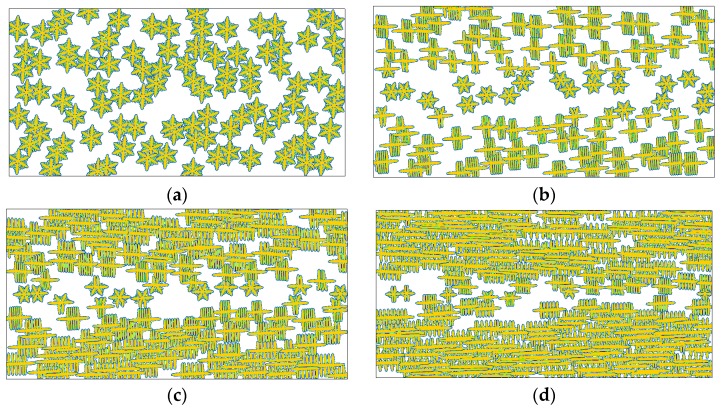
Simulated crystallization morphologies in the crystal order parameter field for different flow strength at anisotropy strength ε = 0.03: (**a**) $\hat{\gamma }=1.0,\;t=1000$; (**b**) $\hat{\gamma }=10,\;t=800$; (**c**) $\hat{\gamma }=30,\;t=800$; (**d**) $\hat{\gamma }=100,\;t=600$. Here the time instants are chosen to ensure the results show similar sizes of crystallized regions.

In the variety of polymer crystallizations, the morphological instability causes interfacial patterns that vary between compact and branched, between symmetric and irregular, and between stable and unstable [51]. In phase field modeling, such morphological instability is characterized by the anisotropic surface energy and the interfacial noise. Here, the discussion on anisotropy is given and on noise it is given in a later section. Unlike the results shown in Figure 5, if we consider the anisotropic growth of the crystal interface, the simulated crystal morphology is greatly different (see Figure 7). When the history flow is very weak, dendrite-like snowflakes are obtained throughout the whole channel (as shown in Figure 7a). This morphology is also a typical crystal pattern usually observed under quiescent conditions [28]. However, as the flow strength is increased, oriented crystals, which are first shish-kebab crystals and then fibrillar crystals in the situations considered, appear near the skin layer. The typical shish-kebab structure consists of a fibrillar core oriented along the flow direction (shish) with transverse, periodically stacked lamellae (kebab). The formation of fibrillar crystals may be explained as too many oriented nuclei grow to fibrils near the skin layer and the narrow space among those fibrils limits their lateral growth. Specifically, when the flow strength is big enough, a typical skin-core structure along the thickness direction is observed. That is the crystal morphologies presented from the skin layer to the core layer are fibrillar crystals, shish-kebab crystals, and spherulites. This result agrees with many experimental observations [52], and indicates that flow induced nucleation plays an important role in producing the skin-core structure. Moreover, the area occupied by oriented crystals enlarges with increasing flow strength. So, it can be expected that oriented crystals will appear at the core layer of the channel if the flow strength is sufficiently high. This prediction is consistent with a recent experimental study [53].

**Figure 8:**
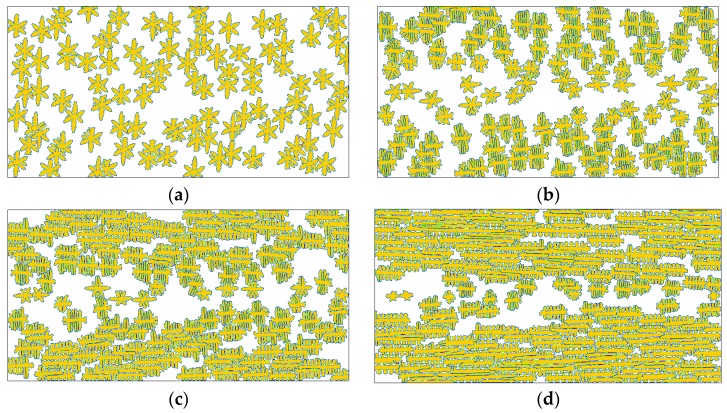
Simulated crystallization morphologies in the crystal order parameter field for different flow strength at anisotropy strength ε = 0.001: (**a**) $\hat{\gamma }=1.0,\;t=2000$; (**b**) $\hat{\gamma }=10,\;t=1600$; (**c**) $\hat{\gamma }=30,\;t=1400$; (**d**) $\hat{\gamma }=100,\;t=1000$. Here the time instants are chosen to ensure the results show similar sizes of crystallized regions.

We have to admit that the anisotropic growth of the crystal interface provides a contribution to the results shown in Figure 7. However, it should be noted that the leading reason for those results is the flow treatment, because even at the very small anisotropy strength ε = 0.001 similar results are also obtained (see Figure 8). In this case, the crystals still show transformation from dendrite-like snowflakes to shish-kebab crystals and fibrillar crystals with increasing flow strength. The main difference between the two cases lies in their microstructures. By comparison, the results shown in Figure 8a present less secondary branches, and as shown in the rest present irregular interfacial patterns. Besides, the reduction of the driving force for branching growth (*i.e.*, anisotropy strength) in Figure 8 slows down the crystallization. 

**Figure 9:**
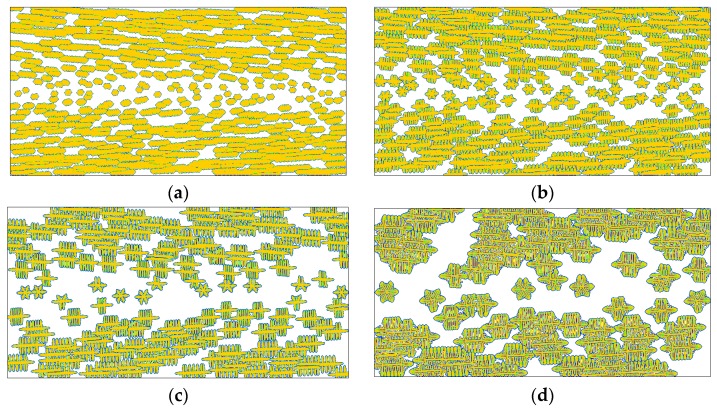
Simulated crystallization morphologies in the crystal order parameter field for the flow strength $\hat{\gamma }=30$ and anisotropy strength ε = 0.03 at different crystallization temperatures: (**a**) *T*_c_ = 180 C, *t* = 200; (**b**) *T*_c_ = 190 °C, *t* = 400; (**c**) *T*_c_ = 200 °C, *t* = 600; (**d**) *T*_c_ = 210 °C, *t* = 1000. Here the time instants are chosen to ensure the results show similar sizes of crystallized regions.

Next, the effects of supercooling on the flow induced crystallization morphology are simply analyzed. Figure 9 shows results obtained for the flow strength $\hat{\gamma }=30$. It is well known that crystallization temperature is one of the most important factors in polymer crystallization. It influences not only the nucleation process but also the crystal growth process. The results shown in Figure 9 embody some of these effects. First, the nucleus number increases with decreasing crystallization temperature. This directly leads to faster crystallization at lower crystallization temperature, and bigger crystal size at higher crystallization temperature. Such behavior is very similar to that existing in quiescent conditions, and there has been some evidence for such behavior in experimental studies [54]. Second, the flow induced crystallization morphologies obtained at different crystallization temperatures differ from each other in microstructures. At the crystallization temperature *T*_c_ = 180 °C, there are many side branches born from the interfaces, but the limited space prevents their growth. On increasing the crystallization temperature, the side branches gain the opportunity to grow. In particular, at the crystallization temperature *T*_c_ = 210 °C, the side branches (kebabs) are even as strong as the backbones (shishes). Despite these differences, the typical features of flow induced crystallization morphologies have not disappeared on changing the temperature. For example, oriented crystals still appear near the skin layer, the length of the oriented crystals decreases along the skin to core direction, orientations of the oriented crystals are approximately parallel to the flow direction, and crystallization near the skin layer is faster than that near the core layer.

In practice, experimental errors and random fluctuations are often unavoidable, and they can lead to unexpected change of the physical process [55]. In simulations, these errors and fluctuations are impossible to be exactly measured, although they really exist. Therefore, noise is introduced to reflect these errors and fluctuations. Because the noise is a comprehensive embodiment of many random factors in physics, we call it physical noise in this paper. In our simulations, the noise reflects the errors and fluctuations from material data, experimental conditions, chain structures, molecular conformations, heat release, *etc.* In phase field simulations, small noise can trigger crystal patterns with plentiful microstructures or even some new patterns, which show better agreement with experimental observations [25,42]. So, in order to make the simulations more realistic, physical noise is added to the growing interface. In this work, we use a uniformly distributed random variable defined on an interval [−*A*, *A*] to describe noise, where *A* is the amplitude of the noise. As with our previous work [56], the noise is introduced to the unstable energy barrier ζ.

**Figure 10:**
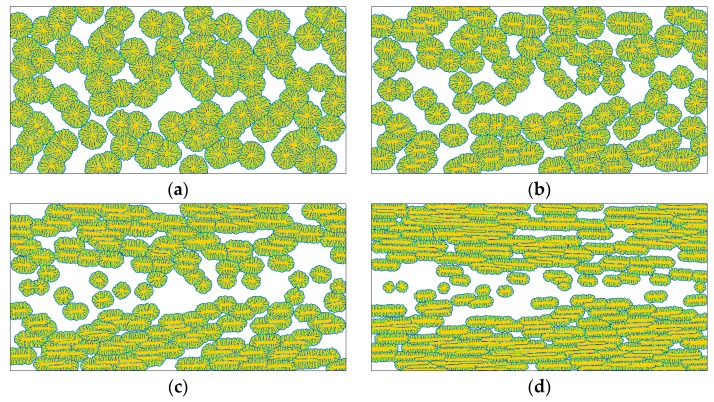
Simulated crystallization morphologies in the crystal order parameter field for different flow strength without considering the interface anisotropy but with introducing a relatively big noise (*A* = 0.1): (**a**) $\hat{\gamma }=1.0,\;t=1000$; (**b**) $\hat{\gamma }=10,\;t=800$; (**c**) $\hat{\gamma }=30,\;t=600$; (**d**) $\hat{\gamma }=100,\;t=400$. Here the time instants are chosen to ensure the results show similar sizes of crystallized regions.

Figure 10 shows some results obtained at ε = 0 and *A* = 0.1. Different from the above simulations, the current simulation condition does not consider the anisotropic growth of the crystal interface, but introduces noise to the crystal interface. It is evident that physical noise has a great influence on the flow induced crystallization morphology compared to the results shown in Figure 5, where both the anisotropy and noise are not involved. Once noise is introduced, the crystals change from compact patterns to branched patterns due to morphological instability. This phenomenon may be explained as follows. During crystal growth, dense side branches with various sizes (triggered by noises) continuously appear at the crystal interface. The limited space does not allow the growth of too many side branches. Therefore, branches are involved in intense competition for survival. In this process, the bigger ones easily gain the opportunity to grow while the smaller ones are more likely to perish, and eventually irregularly branched patterns are observed as in Figure 10. Specifically, the crystals in Figure 10a present dense lamellar branching structures, which are commonly observed in polymer crystallizations under quiescent conditions [57]. In Figure 10b, oriented crystals begin to appear, and this becomes more obvious in Figure 10c,d. Compared with the results obtained in other simulation conditions, the results shown here preserve most properties of the flow induced crystallization morphology. More importantly, these results may be regarded as more realistic than the above ones because they show high agreement with experimental observations [48]. Therefore, we may infer that physical noise in reality plays an important role in generating common flow induced crystallization morphologies.

At the moment, the reader may have noticed that there are two variables in the above comparisons, which are $\hat{\gamma }$ and *t* in Figure 5, Figure 7, Figure 8 and Figure 10, and *T*_c_ and *t* in Figure 9. Generally, we make comparisons by changing the value of one variable and fixing the values of the others for fairness. However, it is hard to carry out absolutely fair comparisons between crystal morphologies. Perhaps there may be two ways for carrying out comparisons. One is to keep the results having similar crystallized regions and the other is to keep them at the same time instant. The first way ensures the results have similar sizes (crystallized regions) but does not ensure that they are obtained at the same time, while the second way is the contrary. Therefore, both ways are not absolutely fair. By comparison, we chose the first way in this study, because it is actually carried out at almost the same crystallization stages. More importantly, it does not impact the objectivity of our conclusions due to the following two reasons. First, time evolution during crystallization does not change the features of morphology endowed by flow treatment. Second, we are more concerned with the flow effect on the final crystal patterns (morphologies).

On the whole, the qualitative agreement of the above simulation results with experimental observations existing in the literature, demonstrates the effectiveness of our phase field technique in predicting polymer crystallization morphology under flow induced conditions. It is found that polymer crystallization is rather complex and diverse. The obtained morphology is strongly dependent on multiple factors. It is quite common to obtain crystals with different patterns for the same polymer under the same flow condition. In spite of this, the flow induced morphological features always present in every pattern. Therefore, compared with other factors, flow plays the most important role in producing oriented crystal morphologies. Finally, it should be mentioned that quantitative formulas for several of the factors are unavailable at present, and thus there is still much work to be done to obtain quantitative results.

## 5. Conclusions

In this work, the phase field method for simulating polymer crystallization morphology under quiescent conditions was successfully extended to flow induced conditions. Firstly, in modeling flow induced nucleation, the influences of flow on nucleus shape and nucleus number are both taken into consideration. Concretely, the nucleus shape is related to the flow induced structures and nucleus number is linked to the flow induced stress. Here, the flow induced structures including molecular orientation and stretch are calculated by coupling the FENE-P dumbbell model in the microscale with the viscoelastic Navier-Stokes equations in the macroscale. Secondly, in modeling the subsequent crystal growth upon the flow induced nuclei, a phase field method for polymer crystallization is used. Finally, simulations are carried out for the crystallization of isotactic polystyrene with the injection molding process which can be simplified as a plane Poiseuille flow in 2D. The influences of flow strength, anisotropy strength, crystallization temperature, and physical noise on the obtained morphologies are studied and compared. Results show that flow plays the most important role in producing oriented crystal morphologies and flow affects the morphology mainly by affecting nucleation. Specifically, when the flow is strong enough, flow induced nucleation surpasses quiescent nucleation as the leading part and a nucleus with high aspect ratio appears near the skin layer. Accordingly, the crystal growth rate is significant accelerated by flow and the crystallization morphology transforms from spherulites to oriented crystals, especially near the skin layer. If the anisotropic growth of the crystal interface is considered, dendrite-like snowflakes are obtained throughout the whole channel at weak flow and oriented crystals, which are first shish-kebab crystals and then fibrillar crystals, are obtained near the skin layer at strong flow. It can be expected that oriented crystals appear at the core layer only if the flow strength is sufficiently high. As to the crystallization temperature, faster crystallization at lower temperature and bigger crystal size at higher temperature can be predicted. Besides, despite the differences in microstructures, the key features of the crystallization morphologies induced by flow do not change with temperature. Lastly, more realistic results are obtained when physical noise is introduced to the simulations. Therefore, noise in reality plays an important role in generating common flow induced crystallization morphologies. On the whole, the influences of flow on morphology and kinetics near the skin layer are usually greater than that near the core layer. For this reason, when the flow is strong enough, typical skin-core structures along the thickness direction can be observed. These results demonstrate that the proposed phase field technique has the ability to give qualitative predictions of polymer crystallization morphology under flow induced conditions.
